# Validation of CZECANCA (CZEch CAncer paNel for Clinical Application) for targeted NGS-based analysis of hereditary cancer syndromes

**DOI:** 10.1371/journal.pone.0195761

**Published:** 2018-04-12

**Authors:** Jana Soukupova, Petra Zemankova, Klara Lhotova, Marketa Janatova, Marianna Borecka, Lenka Stolarova, Filip Lhota, Lenka Foretova, Eva Machackova, Viktor Stranecky, Spiros Tavandzis, Petra Kleiblova, Michal Vocka, Hana Hartmannova, Katerina Hodanova, Stanislav Kmoch, Zdenek Kleibl

**Affiliations:** 1 Institute of Biochemistry and Experimental Oncology, First Faculty of Medicine, Charles University, Prague, Czech Republic; 2 Centre for Medical Genetics and Reproductive Medicine, Gennet, Prague, Czech Republic; 3 Department of Cancer Epidemiology and Genetics, Masaryk Memorial Cancer Institute, Brno, Czech Republic; 4 Research Unit for Rare Diseases, Department of Paediatrics and Adolescent Medicine, First Faculty of Medicine, Charles University and General University Hospital in Prague, Prague, Czech Republic; 5 Department of Medical Genetics, AGEL Laboratories, AGEL Research and Training Institute, Novy Jicin, Czech Republic; 6 Institute of Biology and Medical Genetics, First Faculty of Medicine, Charles University and General University Hospital in Prague, Prague, Czech Republic; 7 Department of Oncology, First Faculty of Medicine, Charles University and General University Hospital in Prague, Prague, Czech Republic; German Cancer Research Center (DKFZ), GERMANY

## Abstract

**Background:**

Carriers of mutations in hereditary cancer predisposition genes represent a small but clinically important subgroup of oncology patients. The identification of causal germline mutations determines follow-up management, treatment options and genetic counselling in patients’ families. Targeted next-generation sequencing-based analyses using cancer-specific panels in high-risk individuals have been rapidly adopted by diagnostic laboratories. While the use of diagnosis-specific panels is straightforward in typical cases, individuals with unusual phenotypes from families with overlapping criteria require multiple panel testing. Moreover, narrow gene panels are limited by our currently incomplete knowledge about possible genetic dispositions.

**Methods:**

We have designed a multi-gene panel called CZECANCA (CZEch CAncer paNel for Clinical Application) for a sequencing analysis of 219 cancer-susceptibility and candidate predisposition genes associated with frequent hereditary cancers.

**Results:**

The bioanalytical and bioinformatics pipeline was validated on a set of internal and commercially available DNA controls showing high coverage uniformity, sensitivity, specificity and accuracy. The panel demonstrates a reliable detection of both single nucleotide and copy number variants. Inter-laboratory, intra- and inter-run replicates confirmed the robustness of our approach.

**Conclusion:**

The objective of CZECANCA is a nationwide consolidation of cancer-predisposition genetic testing across various clinical indications with savings in costs, human labor and turnaround time. Moreover, the unified diagnostics will enable the integration and analysis of genotypes with associated phenotypes in a national database improving the clinical interpretation of variants.

## Introduction

Hereditary cancer syndromes are heterogeneous diseases characterized by the development of various cancer types in carriers of rare germline mutations in cancer susceptibility genes. These genes dominantly code for tumor suppressor proteins negatively regulating mitotic signals and cell cycle progression, activating apoptotic pathways, or executing DNA repair processes [1].

In general, it is considered that around 5% of all cancer diagnoses arise in hereditary cancer form. However, the percentage of hereditary cancers varies by cancer type, ranging from less than 3% in lung cancer to over 30% in pheochromocytoma [2, 3]. Important features distinguishing hereditary and sporadic cancers include an increased lifetime cancer risk with early disease onset, an increased risk of cancer multiplicity, the accumulation of cancer diagnoses in affected families, and a 50% risk of disease trait transmission to the offspring [1]. Considering these attributes and their consequences in terms of decreased life expectancy, decreased quality of life and increased medical expenses, patients carrying mutations in cancer susceptibility genes and their relatives represent a medically important subgroup with specific needs for increased cancer surveillance, a tailored follow-up and therapy, and rational prevention. However, the primary need is an unequivocal identification of the causative germline variant.

Although cancer inheritance has been suggested for over 150 years, the first gene conferring an increased cancer risk (*Rb*) was discovered only 30 years ago [4]. Hundreds of predisposing or candidate genes have been characterized since then, including the clinically most important “major” cancer susceptibility genes with high penetrance representing a subset of genes whose germline variants confer a high cancer risk (with relative risk (RR) > 5.0) in a substantial proportion of hereditary cancer patients. Pathogenic germline variants in “major” genes occur most commonly in patients with breast, ovarian, and colorectal cancers with variable proportions across populations worldwide. The group of cancer susceptibility genes with moderate penetrance is more extensive and growing steadily [5]. However, the clinical utility for many moderate penetrance genes is currently limited by the insufficient evidence about the degree of cancer risks associated with their germline variants.

The rapid improvement and availability of next-generation sequencing (NGS) technologies enable efficient simultaneous analyses of many cancer susceptibility genes in oncology patients or asymptomatic individuals at risk in routine diagnostics. NGS offers multiple approaches for the investigation of cancer predisposition, including the sequencing of whole genomes, exomes or transcriptomes. At present, however, the most widely used method of detecting clinically informative genetic alterations in the clinical setting is targeted panel NGS, analyzing selected subsets of genes of interest [6]. Nevertheless, the numbers of genes included in panels differ substantially among laboratories and depend on healthcare systems. While some cancer-specific or multi-cancer panels include only the “major” predisposition genes for which substantial literature exists with regard to their diagnostic relevance, others include larger gene sets consisting of all clinically relevant genes and additional genes for which the evidence of cancer predisposition is still unclear.

NGS-based cancer testing has been rapidly adopted by routine clinical laboratories [7]. Their primary choice resides in the decision whether to use a commercially available NGS panel, or to design custom-made systems. The decision is influenced by clinical demand determining the set of targeted genes, by the spectrum of cancer diagnoses that will be analyzed, by the expected number of analyzed samples, and by costs of the analyses.

Our aim was to develop a universal diagnostic approach suitable for contributing genetic laboratories and allowing sample batching across multiple cancer indications. We focused on i) designing a custom-made multi-cancer panel with the desired sequencing quality and uniformity permitting a reliable variant identification, ii) the development of a robust analytical procedure limiting inter-run and inter-laboratory differences, and iii) the optimization of the bioinformatics pipeline enabling unified variant calling and annotation. The data collected from analyses of high-risk individuals performed in contributing laboratories will be used to create a nationwide genotype–phenotype database improving clinical variant interpretation in high-risk individuals.

## Methods

### Validation samples

#### Patient DNA samples

Validation of CZECANCA pipeline included analyses of 389 samples previously tested for the presence of germline variants available from DNA repository of the Institute of Biochemistry and Experimental Oncology. First Faculty of Medicine, Charles University. Of these, 137 samples carried pathogenic SNVs or short indels (in *BRCA1/2*, *PALB2*, *CHEK2*, *ATM*, *NBN*, *DPYD*, *PPM1D*, *RAD51C*, *RAD51D*, or *TP53*), 217 had been tested negatively using previous gene-by-gene analyses based on Sanger sequencing or a protein truncation test (PTT) [8–16], and 35 samples carried intragenic rearrangements in *BRCA1*, *CHEK2*, *PALB2*, or *TP53*, identified by the MLPA (multiplex ligation-dependent probe amplification) analysis [10, 17, 18]. All blood-isolated DNA samples were obtained from individuals that gave their written informed consent with mutation analyses of cancer susceptibility genes and who agreed to use their genetic material for research purposes. The study was approved by Ethics Committee of the First Medical Faculty, Charles University and General University Hospital in Prague. All used samples were anonymized prior analysis.

#### Human genome reference standards

Five commercially available DNA reference standards (NA12878, NA24149, NA24385, NA24631 and NA24143) were obtained from Coriell Institute for Medical Research. Well described genotypes, including high confident calls for variant and wild-type alleles, is the major advantage of these reference standards. The genotypes and variants in reference samples identified by CZECANCA analysis and obtained from reference variant-call format (VCF) files (available from the Genome in a Bottle (GIAB) website; http://jimb.stanford.edu/giab/), respectively, were compared to compute CZECANCA sensitivity, specificity, and accuracy, as described by Hardwick et al. [19].

### Panel design

The multi-cancer panel CZECANCA was designed using the online NimbleDesign software utility (NimbleGen, Roche; http://sequencing.roche.com/products/software/nimbledesign-software.html). For enrichment, we selected genes with a known predisposition for hereditary breast, ovarian, colorectal, pancreatic, gastric, endometrial, kidney, prostate and skin cancers, together with known DNA repair genes associated (or potentially associated) with cancer susceptibility (a list of 219 selected genes is provided in S1 Table), considering the results of our previous NGS analysis with a broad panel of 581 genes [20]. The primary gene target for probe coverage was represented by all exons (in case of known cancer susceptibility genes) or all coding exons (in other genes), including 10 bases from adjacent intronic regions. The design considered all transcription variants of selected genes available at UCSC website (https://genome.ucsc.edu/; accessed 2015-05-21). The promoter regions of the *BRCA1* and *BRCA2* genes were included into the primary target. The probes were designed using *continuous design* under strict conditions–minimal and maximal *close matches* (number of times in which a probe sequence matches the genome with either ≤ 5 insertions or deletions, or gap of ≤ 5 bp) were one and three, respectively, allowing us to hybridize the probes up to three targets across the genome. Because of the strict design conditions, some clinically relevant regions were left untargeted for technical reasons such as repeats and homologous regions (see S1 Table). The final panel target size reached 628,069 bases.

### Library preparation

Five hundred ng of genomic DNA isolated from peripheral blood and dissolved in TE buffer was used for preferred ultrasound shearing using Covaris E220 (Covaris Inc). As an alternative DNA fragmentation method, we tested enzymatic digestion using Fragmentase (KAPA Biosystems, Roche) with incubation for 25 min at 37°C according to the manufacturer’s instruction. The mean average size of DNA fragments targeted 200 bp. Sizing and quality was controlled using the Agilent High Sensitivity DNA kit on the Agilent 2100 Bioanalyzer System (Agilent).

Libraries were prepared using the KAPA HTP Library Preparation kit (for ultrasound-sheared DNA samples) or KAPA HyperPlus Kit (for Fragmentase-digested DNA samples) according to the manufacturer’s instructions (KAPA Biosystems, Roche) with minor modifications including the use of universal in-house prepared adapters, double-indexing primers for ligation-mediated polymerase chain reaction (LM-PCR), and primers for post-capture PCR, as described further. The adapters [Adapter#1: 5’-ACACTCTTTCCCTACACGACGCTCTTCCGATC*T-3’ (“*” denotes for phosphothiolate bond) and Adapter#2: 5’-pGATCGGAAGAGCACACGTCTGAACTCCAGTCAC-3’ (“p” denotes for 5’ phosphate)] were hybridized in Tris:NaCl buffer mix (50 mM Tris:HCl pH 7.5; 50 mM NaCl) in 97°C for 2 min, followed by 72 cycles involving incubation at 97°C for 1 min (-1°C per cycle) and 25°C for 5min. The barcoding of size-selected DNA fragments enabling subsequent sample pooling was performed during LM-PCR with indexing primers [Primer#1: 5’-AATGATACGGCGACCACCGAGATCTACACxxxxxxxxACACTCTTTCCCTACACGACGCTCTTCCGATC*T-3’ and Primer#2: 5’-CAAGCAGAAGACGGCATACGAGATxxxxxxxxGTGACTG GAGTTCAGACGTGTGCTCTTCCGAT*C-3’ (“*” denotes for phosphothiolate bond; “xxxxxxxx” denotes for a sequence of particular indices same as the Illumina Truseq HT index i7 and i5)]. The number of LM-PCR cycles was reduced to six to limit the presence of PCR duplicates. Sizing and quality after the double-sided size selection and LM-PCR were controlled using the Agilent High Sensitivity DNA kit on the Agilent 2100 Bioanalyzer System.

To reach the targeted mean coverage (100X), 30 individual barcoded samples (33 ng each) were pooled for the enrichment (usually two overnight hybridizations; tested for 16–72 hours without a significant effect on enrichment efficacy) using the CZECANCA (NimbleGen SeqCap EZ Choice, Roche) to create a sequencing library. After the enrichment, the library was amplified using Primer 1: 5’-AATGATACGGCGACCACCGAGATCTACAC-3’ and Primer 2: 5’-CAAGCAGAAGACGGCATACGAGAT-3’. The number of post-capture PCR cycles was reduced to 11 to reach the optimal library concentration (2 ng/μl) and to minimalize the number of PCR duplicates.

After the enrichment control using qPCR (NimbleGen SeqCap EZ Library SR User's Guide), the final 18 pM libraries were sequenced on the MiSeq system using MiSeq Reagent Kit v3, 150 cycles (Illumina).

### Bioinformatics

#### Single nucleotide variants (SNVs)

The NGS data obtained from sequencing with the CZECANCA were processed using an analysis pipeline based on standard tools. FASTQ files were generated by MiSeq. The quality of raw data was controlled using FastQC v0.11.2 (https://www.bioinformatics.babraham.ac.uk/projects/fastqc/). FASTQ files were subsequently mapped using Novoalign v2.08.03 to hg19 (http://www.novocraft.com/products/novoalign/) to generate sequence alignment map (SAM) files. SAM files were transformed to binary form (BAM files) using Picard tools v1.129 (https://broadinstitute.github.io/picard/). Raw BAM files were further processed to eliminate PCR duplicates of mapped reads. The quality of mapped bases was checked and recalibrated according to default settings using Genome Analysis Toolkit (GATK) v3.3 (https://software.broadinstitute.org/gatk/). The finalized BAM file was converted using a GATK pipeline to a variant-call format (VCF) containing alternative variants only. ANNOVAR was used to annotate VCF files generated using GATK [21, 22] and to check the presence of each variant in external databases (ExAC, 1000Genome or ClinVar) [23–25]. Predictive values from selected prediction algorithms (for example SIFT [26], Mutation Analyzer [27], MutationTaster [28], LRT [29], PolyPhen-2 [30], phyloP [31], GERP [32], CADD [33] or spidex (https://www.deepgenomics.com/spidex) were added to the annotated alternative variants.

For a comparison with CZECANCA sequencing, the data from routine analyses using the TruSight cancer panel (Illumina), performed in a laboratory of the Masaryk Memorial Cancer Institute in Brno were analyzed by an identical bioinformatics pipeline [34].

The Integrative Genomics Viewer (IGV) was used for visualization and manual inspection of individual BAM files [35].

#### Medium-size indels

The detection and exact sequence determination of medium-size insertions and tandem duplications (involving approximately half of the sequence reads, depending on the sequencing chemistry used) is very challenging. The identification of these alterations was based on the method of soft-clipped bases using Pindel (http://gmt.genome.wustl.edu/packages/pindel/) [36]. The finalized BAM files served as an input for the analysis. In our case (with mean read size of 75 bp; MiSeq Reagent Kit v3, 150 cycles chemistry) insertion or duplication exceeding 35 bp was considered as a medium-size indel.

#### Copy number variations (CNVs)

An analysis CNVs was performed using the CNVkit (https://pypi.python.org/pypi/CNVkit). The CNVs analysis is coverage-based and therefore required good coverage uniformity. Raw BAM files served as the input for this analysis.

#### Coverage visualization

The visualization of sequence coverage of the individual samples, enabling a fast visual inspection of coverage limit >20X (for a reliable identification of heterozygotes) across the analyzed genes, was performed by an in-house “Boudalyzer” script written in R language. The coverage is visualized from the finalized BAM files. This tool was used for the generation of manuscript figures showing coverages of the analyzed genes.

#### Variant interpretation

We used the scoring scheme outlined in ENIGMA guidelines (https://enigmaconsortium.org/) for variant interpretation to classify SNVs and indels as benign (Class 1), likely benign (Class 2), variant of unknown significance (Class 3), likely pathogenic (Class 4) and pathogenic (Class 5) [37]. Identified variants of unknown significance (VUS) were further prioritized if their minor allele frequency was lower than 1% in ExAC, 1000Genome databases, or in a two sets of population-matched controls containing anonymized genomic data from 530 non-cancer controls analyzed by CZECANCA NGS and from 780 unselected Czech individuals analyzed by an exome sequencing (provided by the National Center for Medical Genomics; http://ncmg.cz). Potentially deleterious VUSes were selected based on concordant results obtained from above-mentioned *in silico* prediction algorithms. These priorized VUS variants were enrolled into the list of variants for subsequent segregation analyses or functional *in vitro* testing performed in selected genes.

The CZECANCA contains 22 genes that are listed in the ACMG recommendation (S1 Table) for the reporting of secondary findings [38].

## Results

### Target gene coverage

**Fig 1:**
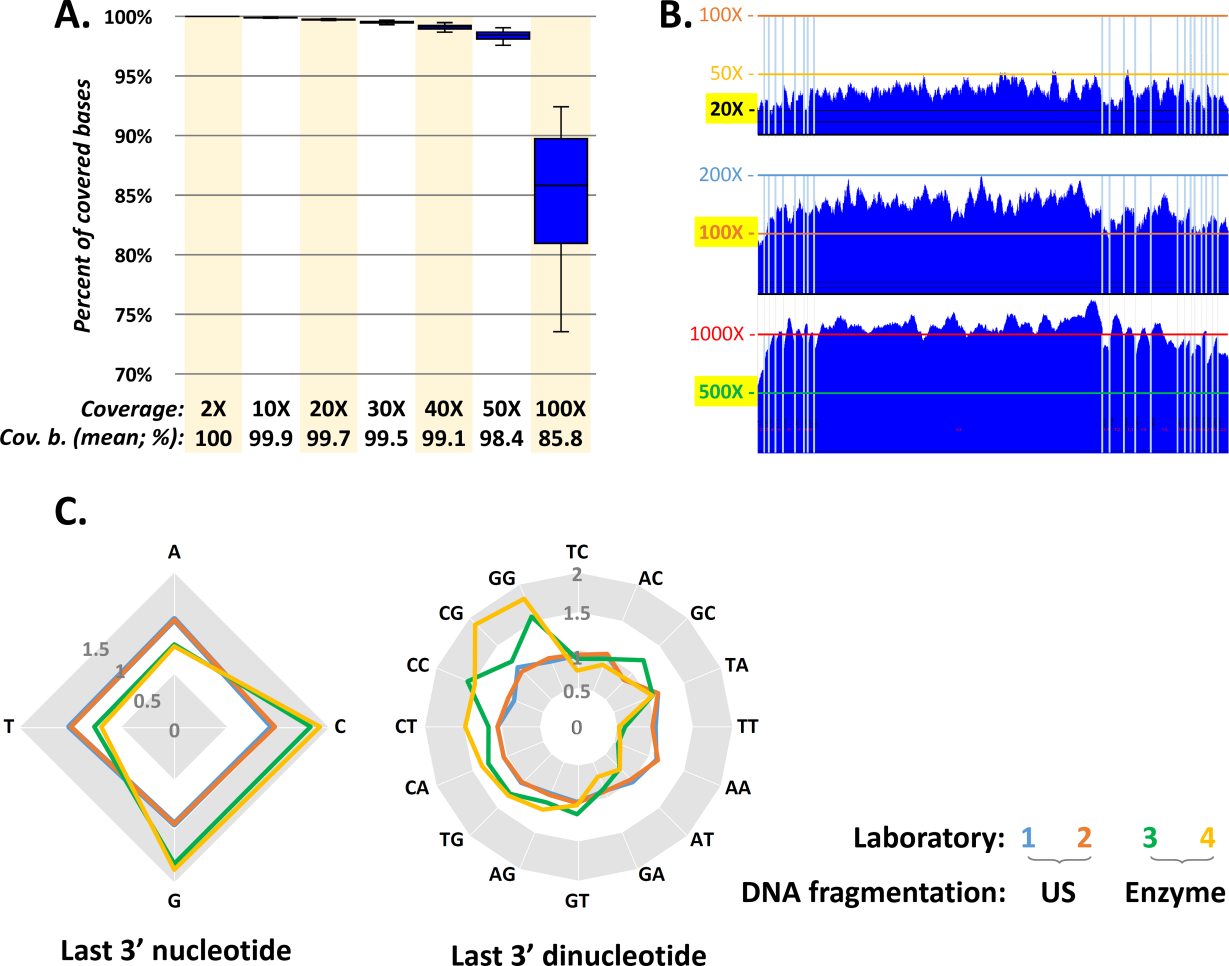
Coverage parameters from CZECANCA sequencing. (A) The chart expresses the percentages of covered target bases (cov. b.) obtained from 25 analyzed samples from a standard run targeting sequencing coverage 100X. (B) The coverage (at y-axis) of *BRCA1* coding sequence (NM_007294; x-axis; vertical lines represent exon boundaries) in three independent runs targeting sequencing coverages 20X, 100X, or 500X demonstrates coverage uniformity, not influenced by coverage depth. (C) The “randomness” of the DNA shearing approach using ultrasound (US) and enzymatic cleavage was compared by an analysis of the distribution of ending nucleotides and dinucleotides in reads completely mapped to the large exon 11 (chr17:41243452–41246877; 3426bp) in the *BRCA1* gene, representing one of the largest continuous genomic fragments targeted by CZECANCA probes. The chart displays the relativized distribution of terminal nucleotides and dinucleotides in the analyzed region from 12 samples from each laboratory normalized to the average nucleotide and dinucleotide content of the analyzed region. The distribution of last nucleotides and dinucleotides in fragments from samples processed by US oscillate closer to a normalized value (1) than in fragments of samples prepared by the enzymatic cleavage.

**Fig 2:**
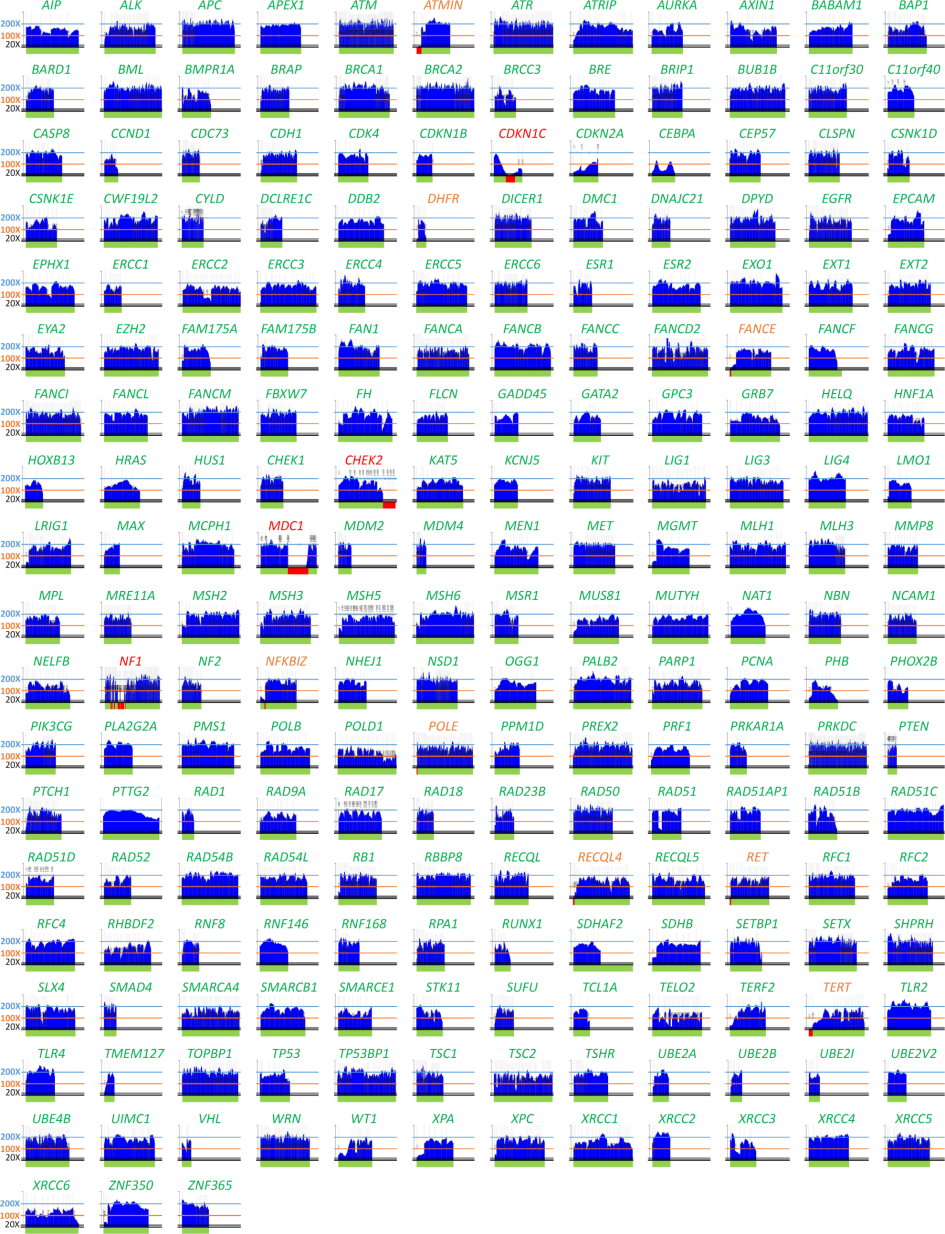
Coverage (y-axis) of coding sequences (x-axis) of 219 CZECANCA target genes from a routine, randomly selected run targeting 100X coverage. Note: Fully covered genes are depicted in green letters, genes with coverage <20X in a single exon are in orange letters, and genes with uncovered regions exceeding single exon or >10% of coding sequence are in red letters. Green horizontal bars (below individual graphs constructed using “Boudalyzer” script) indicate coverage ≥ 20X; red horizontal bars indicate regions covered <20X and uncovered regions.

The NGS analysis with CZECANCA targeting the coding sequences of 219 genes (S1 Table) displayed high coverage uniformity. Under standard conditions for routine analyses, we targeted sequencing coverage 100X. In these settings, more than 85% of the targeted regions were covered 100X, 98% of the targeted regions were covered at least 50X and less than 0.2% of targeted regions had coverage below 20X (Fig 1A). The entire coding sequence was fully covered at least 100X in 144/219 targeted genes (65.8%), at least 50X in 190/219 genes (86.8%), and at least 20X in 207/219 targeted genes (94.5%; Fig 2). Coverage did not exceed 300X in any of the captured targets.

**Fig 3:**
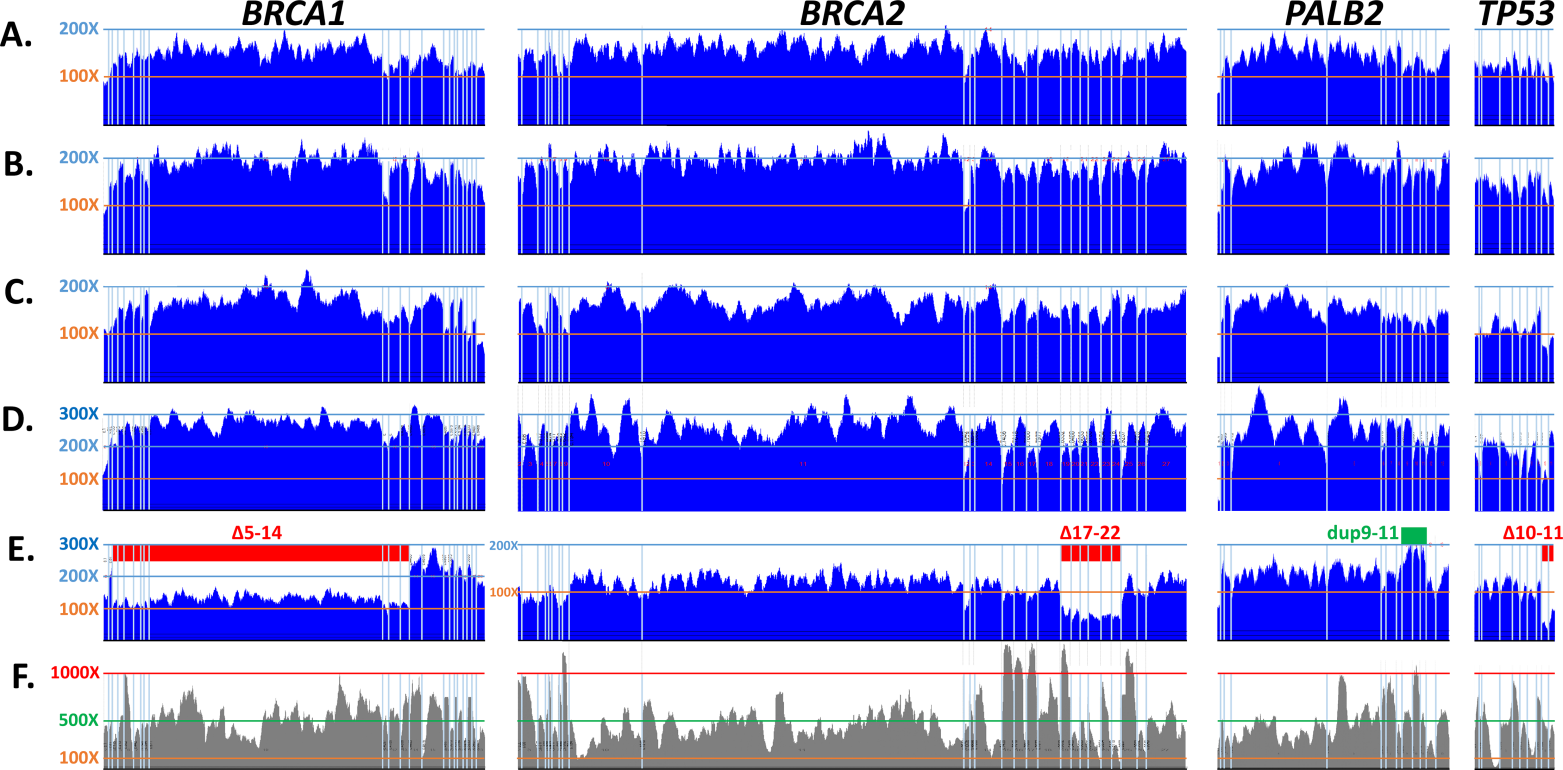
**Coverage of selected genes from the CZECANCA (A-E) and TruSight Cancer sequencing (F) panels.** The pictures show coverage (at y-axis) alongside the coding sequences of *BRCA1* (NM_007294), *BRCA2* (NM_000059), *PALB2* (NM_024675), and *TP53* (NM_000546), the vertical lines represent exon boundaries. Panels A–D show results obtained from a CZECANCA NGS analysis of various samples performed in four participating laboratories using the ultrasound (A, B) or enzymatic (C, D) DNA fragmentation protocol. Examples of the identified CNV aberrations in the depicted genes (deletions in *BRCA1*, *BRCA2* and *TP53* and duplication in *PALB2*) are shown in panel E. For comparison, panel F demonstrates the uneven coverage of the depicted genes by sequencing using the TruSight Cancer panel (Illumina).

Coverage was uniform among samples independently analyzed in the participating laboratories using the described protocol (Fig 3), and also among samples sequenced using separately-synthesized CZECANCA lots (data not shown). The equal coverage uniformity was independent of coverage depth (Fig 1B). The coverage uniformity was partially influenced by the DNA fragmentation approach with better results obtained by ultrasound fragmentation in comparison with enzymatic DNA cleavage. The improved results (more random DNA shearing) obtained with the ultrasound fragmentation protocol were indicated by an analysis of terminal (di)nucleotides in reads from samples prepared by both DNA fragmentation methods, regardless of the laboratory site (Figs 1C and 3). The CZECANCA coverage uniformity substantially surpassed that of the Illumina TruSight Cancer Panel (Fig 3F).

Low-covered regions (uncovered or with coverage ≤20X) were constantly observed in 12/219 genes (5.5%; Fig 2, S1 Table). In nine genes, the low–covered regions were mostly limited to a single exon (typically the first exon) representing usually a small fraction of the coding sequence. In three incompletely covered genes (*CHEK2*, *MDC1*, *NF1*), single or several exons were omitted from the CZECANCA design (see Panel design in Methods). The remaining low-covered regions were GC-rich regions with mean GC content of 76.88% (S2 Table) while the average GC content of the CZECANCA targets is 47%.

Sequencing quality was partially influenced by the particular MiSeq sequencer. In standard runs, more than 99% of bases reached a Phred score >20 (i.e. 99% accuracy) and approximately 97% of bases overcame a Phred score of 30 (i.e. 99.9% accuracy). A decrease in PCRs cycles during library preparation reduced the number of PCR duplicates, which finally represented 7–9% of reads. The mean off-target (reads mapped to distance exceeding 250 bp from the nearest bait) across the performed runs was constantly less than 12% of reads.

### Reproducibility, specificity and sensitivity analysis

The reproducibility of variant calls was tested using intra-, inter-run, and inter-laboratory replicates. During the sequencing of intra-run replicates, we also evaluated the impact of coverage depth on coverage uniformity and reproducibility.

**Fig 4:**
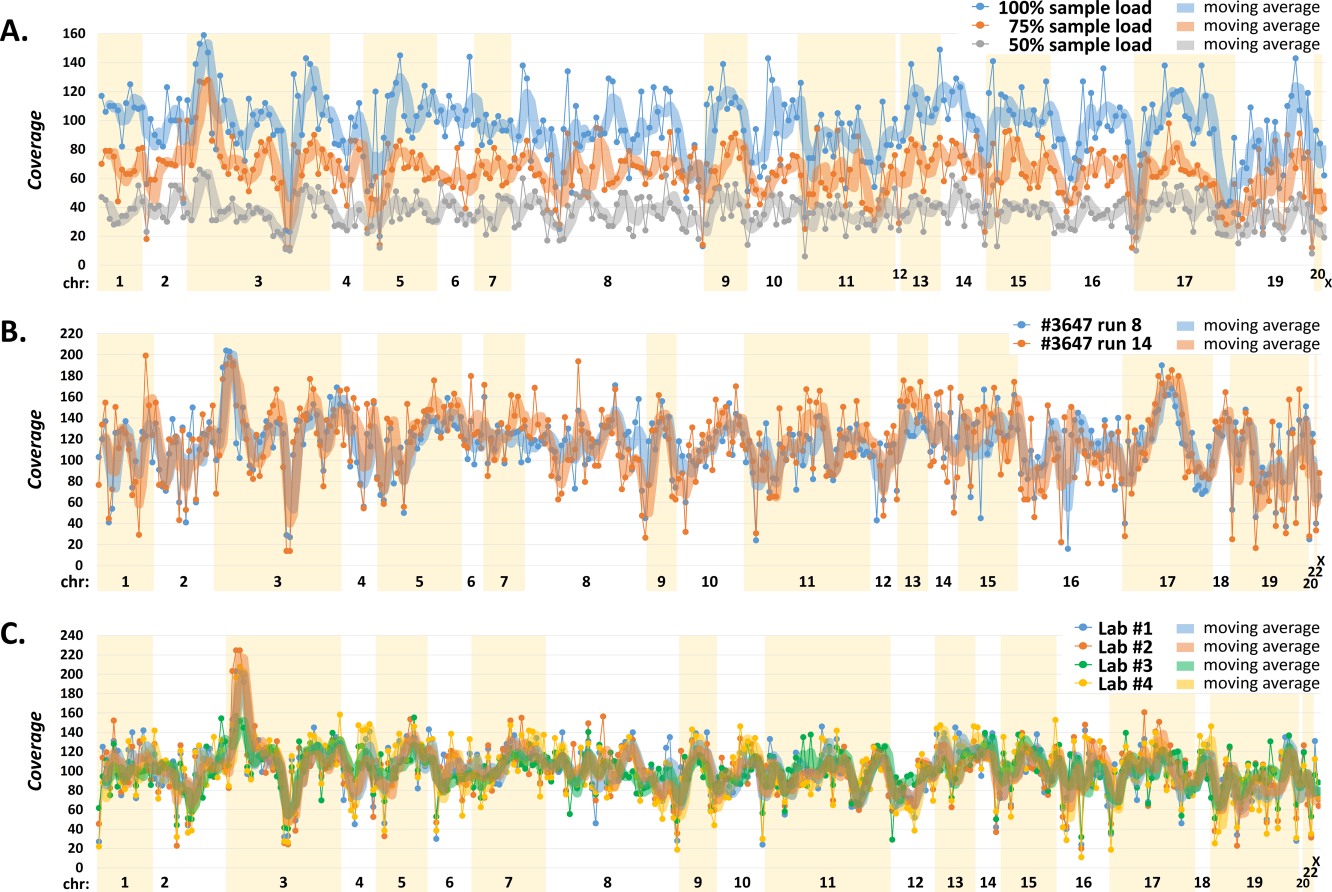
**Analysis of intra-run (A), inter-run (B), and inter-laboratory (C) replicates.** The panels show sequencing coverages (y-axis) of the identified variants arranged according to chromosomal localizations (x-axis). We used moving average curves (average of 3 values) to compare trends in coverages. Panel (A) describes the results of an analysis of three independently processed intra-run replicates from an identical DNA sample pooled in 33 ng (considered as 100%), 24.75 ng (75%), and 16.5 ng (50%), respectively. Panel (B) demonstrates variant coverages identified in two independent inter-run (run 8 and 14) replicates. All coverage values of sample #3647 in run 14 were corrected by a factor of 1.3880 to normalize coverages between samples (see S4 Table). Panel (C) shows coverages of variants identified in an inter-laboratory control sequenced in four laboratories (Lab) participating in panel validation (see S5 Table). The coverages of variants identified in Lab 2, 3, and 4 were normalized to the average coverage of Lab 1 for better comparisons of coverages.

Three individually bar-coded replicates were pooled for enrichment in amounts corresponding to 33 ng (considered as 100%), 24.75 ng (75%), and 16.5 ng (50%), respectively. The subsequent bioinformatics of these samples, considering variants with GATK quality >100 in the targeted regions (exon sequences with 12 bp from adjacent introns), revealed 293 (100%), 292 (99.7%) and 290 (99.0%) variants, respectively (S3 Table). Altogether, 289/293 (98.6%) variants were identified in all replicates, while four variants not detected in DNA-reduced samples were variant homozygotes located in low-covered regions or had GATK quality <100. The analysis demonstrated that alternative nucleotides could still be reliably detected in samples with reduced overall coverage, showing the robustness of the analysis in samples with unequal DNA input (Fig 4A).

A subsequent analysis of inter-run replicates (performed with another DNA sample analyzed in two independent runs) revealed 356 unique variants with GATK quality >100 in at least one replicate (S4 Table). Overall, 354 (99.4%) variants were identified in both inter-run replicates with a strong coverage correlation (Fig 4B).

In addition, the inter-laboratory performance was tested by an NGS analysis of an identical DNA control sample in four laboratories participating in the panel validation (Fig 4C), which revealed 332 unique variants with GATK quality >100 in at least one laboratory, from which we identified 331 (99.7%), 327 (98.5%), 329 (99.1%), and 329 (99.1%) variants in the particular laboratory, respectively. The discordant findings were caused by variants in low-covered regions, with low base Phred quality, or GATK quality <100 (S5 Table).

Sensitivity and specificity were assessed in 354 samples previously tested for the presence of germline variants. All 137 previously identified pathogenic germline mutations in *BRCA1/2* and other susceptibility genes were detected by CZECANCA (S6 Table). Moreover, an analysis revealed nine additional *BRCA1* or *BRCA2* mutations. Of these, seven mutations were identified in samples previously tested by cDNA sequencing (they had not been detected previously, probably because of nonsense-mediated decay). The pathogenic missense mutation c.3G>A in *BRCA2* was found in a sample negatively analyzed using PTT and the pathogenic *BRCA2* mutation c.5645C>A was found in the carrier of c.5266dupC in *BRCA1* in whom the identification of a pathogenic *BRCA1* variant discontinued subsequent *BRCA2* testing.

**Fig 5:**
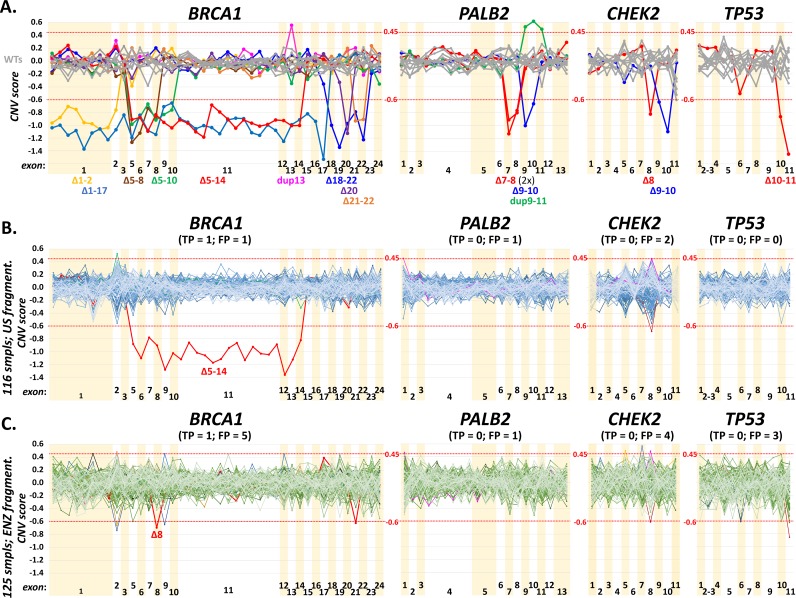
**The panel A show results of CNV analysis revealing large deletions or duplications in four genes in a testing set of 35 samples with previously identified CNVs.** The charts show median-normalized values of CNV scores for particular gene bins (default settings in CNVkit software; S8 Table). Values <-0.6 and >0.45 (red dotted lines) were assumed as thresholds indicating a deletion or a duplication, respectively. All shown CNVs were confirmed by MLPA previously (S7 Table). The panels B and C demonstrate frequency of true positive (TP) and false positive (FP) CNV signals from analyses performed in two participating laboratories (laboratory 1 in B and laboratory 3 in C). While 116 samples analyzed in four consecutive runs in B were prepared using the ultrasound (US) fragmentation, 125 other samples in four consecutive runs in C were prepared using the enzymatic (ENZ) fragmentation method. Samples in vivid colors highlight suspected samples that were further analyzed by MLPA analysis and samples in *BRCA1* Δ5–14 (B) and Δ8 (C) denote for true positives. The presence of putative CNVs in *PALB2*, *CHEK2*, and *TP53* were excluded by analysis that revealed heterozygotes in regions with suspected deletions or by an MLPA analysis.

Further, we validated the sensitivity of CNVs detection on 35 samples tested positively using the MLPA analysis (S7 Table). All CNVs including 18 samples with large *BRCA1* deletions or duplications, 12 CNVs in *CHEK2*, four in *PALB2* and one in *TP53* were detected using CNVkit software in routine settings targeting 100X coverage (Fig 5A; S8 Table). This analysis also enabled to setup CNVkit thresholds indicating the presence of a deletion or a duplication. To estimate the number of false positive and true positive CNV calls obtained from CNVkit, we further analyzed aggregated results from four consecutive runs performed in two participating laboratories preparing sequencing libraries by ultrasound shearing and enzymatic digestion, respectively (Fig 5B and 5C). The CNV analysis in *BRCA1* gene revealed that two out of 116 (1.7%) ultrasound-sheared samples (from laboratory 1) and five out of other 125 (4%) enzymatically-digested samples (from laboratory 3) were scored as the samples with suspected deletion or duplication. The *BRCA1* MLPA analysis performed in all samples revealed that one suspected sample from each laboratory was true positive (exon 5–14 del in laboratory 1 and exon 8 del in laboratory 3), remaining suspected samples (one from laboratory 1 and four from laboratory 3) were false positive, and 114/116 in laboratory 1 and 120/125 in laboratory 3 were true negative *BRCA1* samples.

**Fig 6:**
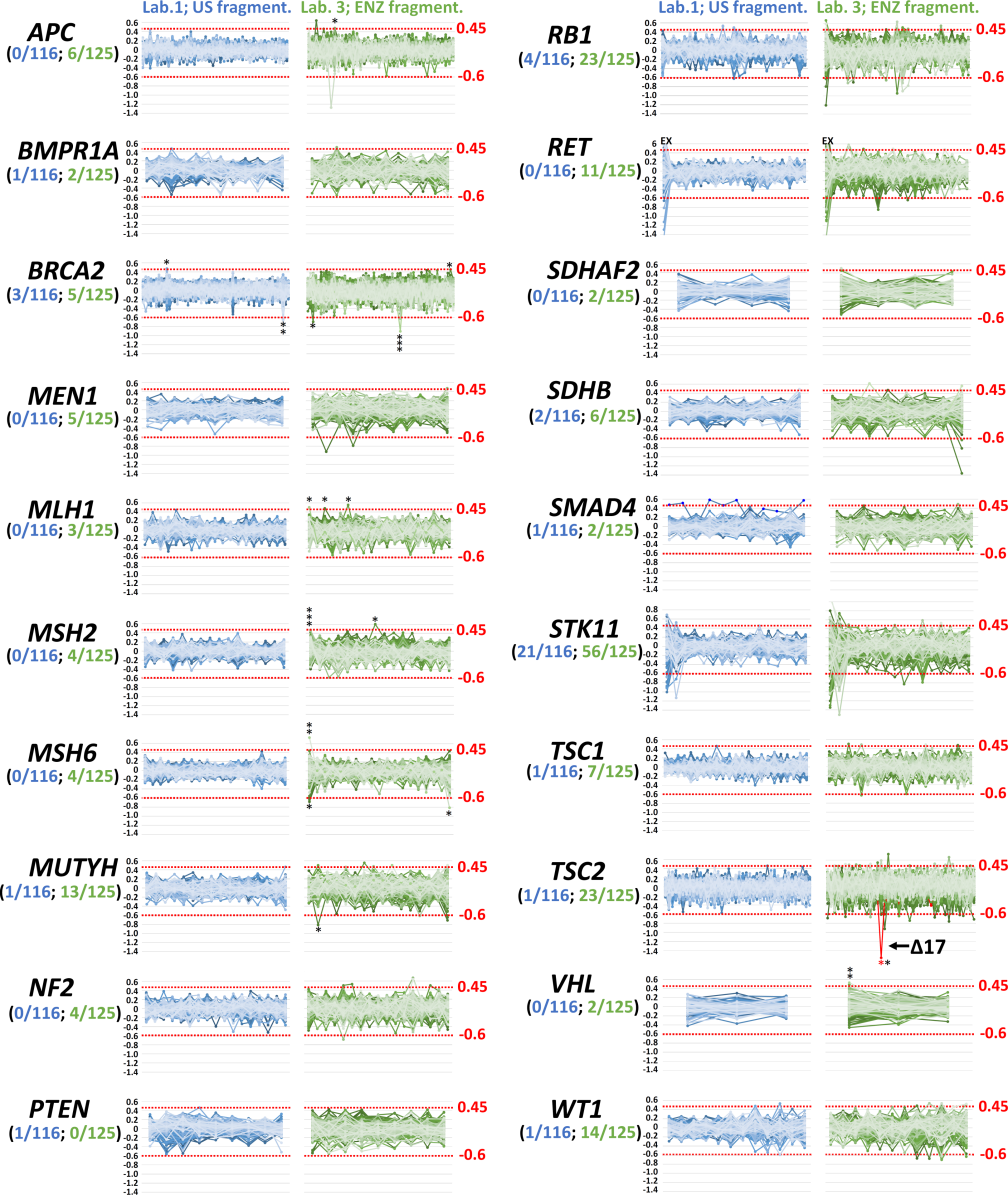
CNV detection is influenced by a DNA preparation method. Panels show analyses of remaining ACMG genes (not shown in Fig 5B and 5C) from four runs performed in laboratory 1 (116 DNA samples fragmented by ultrasound) and laboratory 3 (125 DNA samples fragmented enzymatically). The numbers in parentheses express number of samples with possible CNVs from all analyzed samples in contributing laboratories. *indicate samples analyzed by MLPA negatively (FP–black) or positively (TP–red). Bin set covering exon 1 in *RET* was excluded from the analysis due to the large coverage variability.

While the minimum coverage for a reliable detection of SNVs was estimated at 20X, the minimum coverage required for a reliable detection of CNVs is higher [39]. However, we have noticed that coverage uniformity is at least of the same importance. While the type of the DNA fragmentation protocol (ultrasound vs. enzymatic digestion) did not influence the sensitivity of SNVs detection (Fig 4C), enzymatic digestion caused difficulties in reliable CNVs detection (with an increased number of CNVkit false positives) when comparing samples with the same coverage. We suppose that the main problem of a CNVs coverage-based analysis of enzymatically fragmented samples is worse coverage uniformity caused by non-random DNA cleavage, as discussed above (Fig 1C). To evaluate the sensitivity of CNVs detection in other targeted genes and to better address the influence of DNA fragmentation protocol on the CNV analysis, we compared results of CNVkit analysis in remaining 20 ACMG genes (except *BRCA1* and *TP53* discussed above) covered by CZECANCA target (Fig 6).

The analysis revealed relative low rate of suspected CNVs (0–4 and 0–23 carriers per gene in samples prepared by ultrasound DNA fragmentation and enzymatic DNA digestion, respectively) and demonstrated that preparation of sequencing libraries using ultrasound digestion substantially decreased the need for subsequent MLPA analyses. With the exception of *BRCA2* in which MLPA analysis was performed in all suspected samples, application of MLPA analysis in remaining genes were directed by the phenotype characteristics of analyzed probands. The only CNV identified in remaining ACMG genes was exon 17 deletion in the tuberin (*TSC2*) gene in a patient with typical skin affections. The CNV analysis of the entire set of CZECANCA target genes is provided in S11 Table. The data indicate that deviations of median-normalized CNVkit values in a run of consecutive bin sets could indicate highly probable presence of a large intragenic deletion or duplication (S1 Fig). The extreme case of such situation provides the analysis of genes localized on X chromosome in male and female probands (S2 Fig) that also demonstrates the dynamic range of analysis in detection of real deletion.

For the detection of medium-size insertions and tandem duplications, we added the Pindel tool to the bioinformatics pipeline in order to identify the 64 bp tandem duplication in *BRCA1* (c.5468-11_5520dup64; NM_007294; Chr17: 41197765–41197830 on Assembly GRCh37) not detected by GATK. The sensitivity of a Pindel analysis was recently confirmed by another GATK-omitted variant, the 38 bp duplication in *CHEK2* (c.845_846+36dup38; NM_007194; Chr22: 29105958–29105995 on Assembly GRCh37), confirmed by Sanger sequencing.

**Fig 7:**
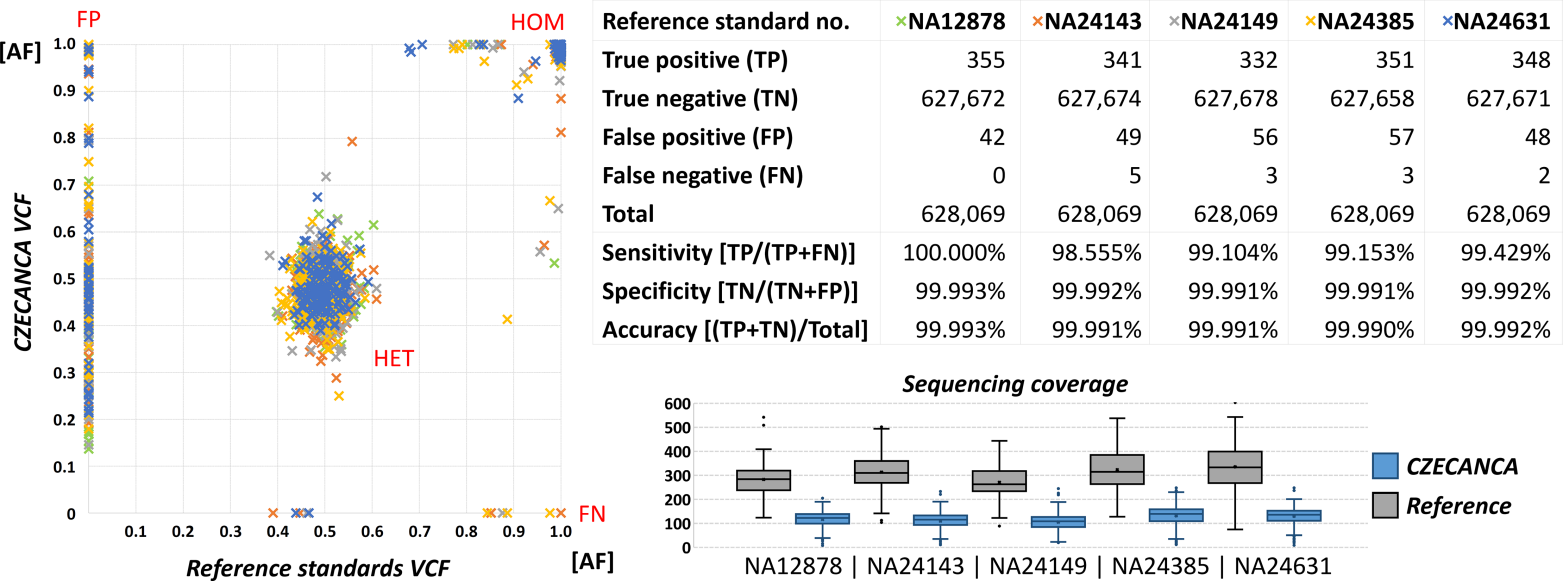
**Comparison of variant detection (shown as values of variant allelic fraction; AF) in DNA reference standards** (NA12878, NA24149, NA24385, NA24631 and NA24143) obtained from CZECANCA analysis (x-axis) and AF from VCF files for these standards downloaded from http://jimb.stanford.edu/giab/ (y-axis). The graph shows all variants with GATK quality >100 reached in CZECANCA analysis (including FP variants) and undetected (FN) variants. Heterozygote variants clustered in the center, while homozygote variants in right upper corner. Variant distribution was partially influenced by the differences in mean sequencing coverage targeting 100X and 300X in CZECANCA and DNA reference standards VCFs, respectively. The number of TP, TN, FP, FN, and total number of variant (= CZECANCA target) was used to calculate of sensitivity, specificity, and accuracy of CZECANCA analysis.

Five DNA reference standards (NA12878, NA24149, NA24385, NA24631 and NA24143) with well-described genotypes were analyzed by CZECANCA pipeline to benchmark the overall workflow performance [19]. Comparison between genotypes identified in CZECANCA analysis and available as reference VCFs showed a high concordance in identification of homozygotes and heterozygotes and also high sensitivity, specificity and accuracy of CZECANCA NGS analysis (Fig 7; S9 Table). Totally, 1,722 true positive variants (332–355 per sample), 252 false positive variants (42–57 per sample), and 13 false negative variants (0–5 per sample) were scored in all analyzed DNA reference standards considering 628,069 bases of CZECANCA target region. All were localized in 84 short genomic regions that comprised in majority homopolymeric or repetitive non-coding sequences creating recurrent sequencing errors in currently used sequencing platforms, as indicated by 7/13 not identified (false negative) variants flanking to position of false positive variants. The subsequent manual IGV inspection revealed that the remaining six false negative variants (all indels) were present with allelic fraction below 15% (filtered out through the bioinformatics pipeline).

Finally, an external quality assessment of CZECANCA was performed using the pilot NGS germline mutations scheme provided by the European Molecular Genetics Quality Network (EMQN; www.emqn.org). This external quality assessment showed a 100% sensitivity of variant detection (S10 Table).

## Discussion

Multi-gene panel NGS has changed the genetic landscape for hereditary cancer syndromes. At present, clinical testing prioritizes the use of smaller cancer-specific panels, usually up to 30 cancer susceptibility genes. A large number of panels is available particularly for breast/ovarian and colorectal cancers, which represent frequent diagnoses with a high contribution of genetic components influencing the disease onset, progression and treatment outcomes [40]. Analyses based on smaller panels mainly simplify the clinical interpretation of the identified genotypes with a reduction of incidental findings. While their use is beneficial in clearly indicated patients with typical phenotype characteristics for a given cancer syndrome, the selection of a proper cancer-specific gene panel is not trivial in individuals with less characteristic features (e.g. patients from multi-cancer families). Moreover, our current knowledge of many cancer syndromes is based on the analyses of mostly prototypical cases, the testing criteria are changing dynamically, and the list of cancer predisposition genes with clinical utility is far less complete. Recently, Pearlman et al. analyzed 450 early-onset colorectal cancer patients and showed that a third (24/72) of mutation-positive patients did not meet the established genetic testing criteria for the gene(s) in which they had a mutation [41]. An analysis of mismatch repair (MMR) genes (traditionally linked to hereditary non-polyposis colorectal cancer) in a set of 34,981 cancer patients in a study by Espenschied et al. revealed that out of 528 patients with MMR mutations, 63 (11.9%) had breast cancer only and thus *MSH6* and *PMS2* mutation carriers may manifest with a hereditary breast and ovarian cancer phenotype [42]. In an analysis of *BRCA1* and *BRCA2* in 1,371 unselected breast cancer cohorts, Grindedal et al. showed that common guidelines identified only 45–90% of mutation carriers [43]. The ultimate solution to identify cancer risks would be an analysis of the whole exome (or even better genome) in all cancer patients; however, the implementation of such a strategy is not realistic at present [44]. We suppose that the use of larger multi-cancer panels (containing hundreds of genes) for an analysis of genetic risk in cancer patients is beneficial for several reasons. i) Such an analysis reveals a complex variation landscape of target genes in different cancers [7]. ii) It reveals carriers of concurrent pathogenic mutations and iii) it enables the testing of affected individuals from multi-cancer families with reasonable costs and turnaround time. Finally, iv) combining all genes of interest in a single panel simplifies and unifies laboratory procedures in a single workflow even if testing for different syndromes.

We have developed the custom-designed CZECANCA multi-cancer panel targeting the coding sequence of 219 cancer susceptibility or candidate genes, enabling the identification of a genetic predisposition in the most frequent hereditary cancer syndromes. Besides the established cancer susceptibility genes, we have decided to include also a subset of genes with low, clinically still unconfirmed utility, although their variants cannot be reported until their clinical evidence is known. These genes code for known interactors of established cancer susceptibility gene products, whose mutations may result in a similar phenotypic outcome. However, we suppose that knowledge obtained through the association of the identified genotypes with the phenotypic characteristics of the analyzed patients may substantially accelerate the process of clinical utility evaluation. Moreover, a subsidiary genetic report could be easily generated from the stored data in case of the approval of new cancer susceptibility genes included in CZECANCA. From the technical point of view, a larger genomic target has a favorable impact on panel complexity, improving its coverage uniformity [45].

The validation of the CZECANCA analytic workflow together with the bioinformatics pipeline is necessary for its implementation into routine diagnostics [46]. The presented analytical workflow was optimized for sequencing using MiSeq Illumina, representing the most frequently used NGS platform currently available in diagnostic laboratories. Genetic testing using gene panels is a cost-effective strategy [47]. The material costs for library preparation and sequencing (chemicals, kits, and disposables) using CZECANCA do not exceed €150 per patient in the standard settings (targeting sequencing coverage 100X). The CZECANCA workflow was intended mainly for medium throughput laboratories. As a universal panel, CZECANCA significantly reduces the turnaround time. The sequencing data for 30 analyzed DNA samples in one sequencing MiSeq run might be available in four days (three days for DNA fragmentation and library preparation, depending on hybridization time, and one day for MiSeq sequencing). We are aware that the low-covered or uncovered regions (affecting 12/219 CZECANCA-targeted genes) may require additional effort and time, when requested for genetic assessment.

The validation showed CZECANCA’s high sensitivity, specificity, analytical robustness, and accuracy. We have demonstrated that SNVs and small/medium-size indels could be detected with high confidence. Moreover, we have shown that the uniform coverage (targeting to mean 100X coverage) of a target sequence enabled a robust identification of CNVs without the need of routine MLPA, serving as the method for independent CNVs confirmation or exclusion of false positivities. However, despite that the number of false positive calls was low and we detect no false negative sample in ACMG genes, we are aware that with caution needs to be interpreted positive CNV calls in genes for which MLPA assay (or other method) are not routinely available for confirmatory purposes. When required, presence of false positive signals can be reduced by the use of ultrasound fragmentation providing unbiased DNA shearing over enzymatic lysis and/or increased sequencing coverage.

**Fig 8:**
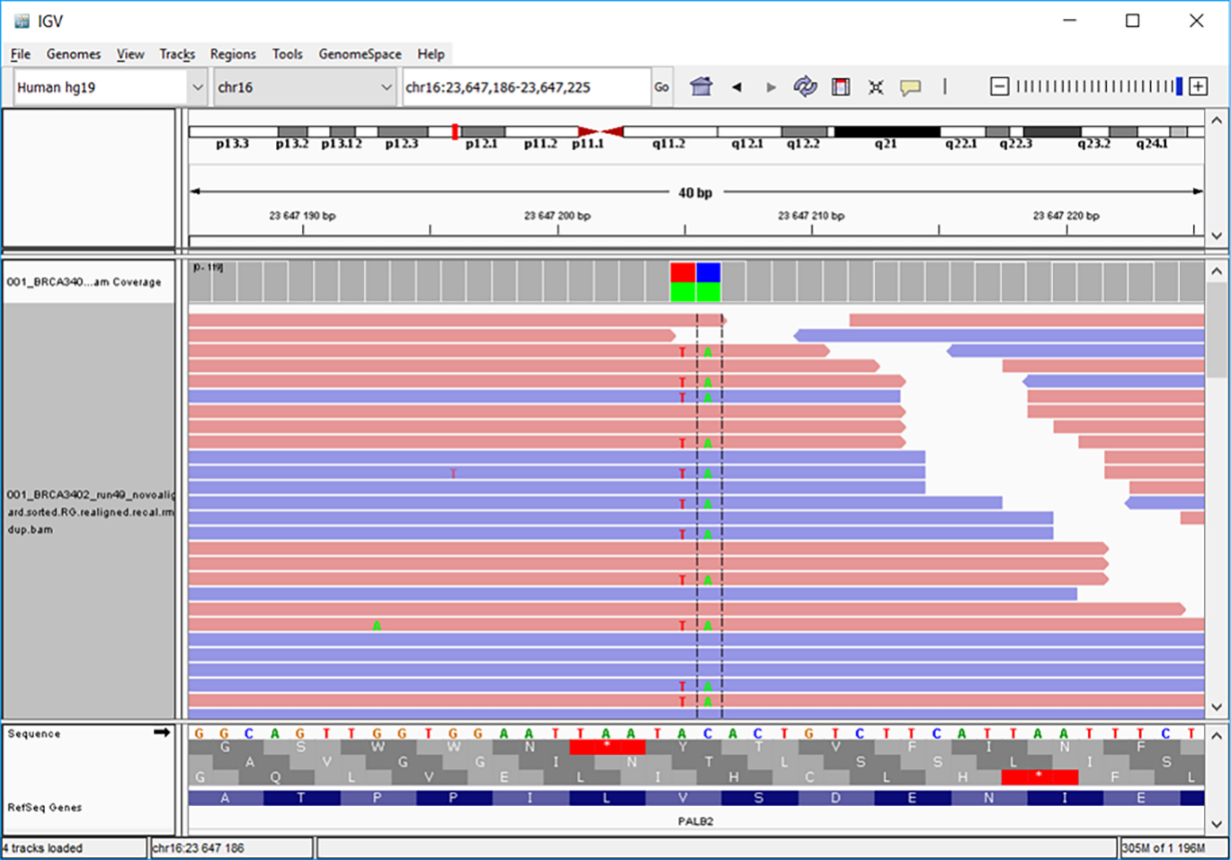
Identification of c.661_662delinsTA double substitution (p.Val221*) in *PALB2* (NM_024675). The BAM file displayed in IGV shows the *cis*-position of both substitutions in approximately 50% of forward (pink bars) and reverse (blue bars) reads, respectively.

Another advantage of NGS (over Sanger sequencing) is its ability to identify *cis* or *trans* positions of compound, closely localized heterozygous SNVs. For example, the position of double substitution in the *PALB2* gene creating a stop codon (c.661_662delinsTA; p.Val221*; NM_024675), which required further analyses (e.g. PTT) before the NGS era [10], can be identified directly from sequencing reads (Fig 8). The identification of additional pathogenic mutations during the validation procedure in negatively pre-tested samples indicated that a re-analysis is warranted for at least high-risk patients negatively tested by historical analyses based on indirect prescreening methods (e.g. PTT) or cDNA sequencing [48].

CZECANCA (CZEch CAncer paNel for Clinical Application) is intended to unify cancer predisposition testing in the Czech Republic, helping diagnostics laboratories transform the gene-by-gene strategy to NGS, even if is not a population-specific panel *per se*. NGS-based technologies bring new challenges including technological aspects, bioinformatics processing, the management of large datasets, and clinical interpretation of results [46]. The use of a uniform analytical and bioinformatics approach improves the identification of technical and platform-specific sequencing errors, as we demonstrated in inter-run and intra-run comparisons. Moreover, validation of the panel using reference standard DNA samples with known genotypes enabled identification of genomic loci (dominantly homopolymeric regions) providing these recurrent sequencing errors, which could be subsequently easily eliminated by bioinformatics. The use of CZECANCA will help generate a global view of constitutional variants from the perspective of known cancer predisposition and candidate genes in the population. Simultaneously with the sequencing of cancer patients, we aim to sequence non-cancer controls in order to identify and establish the frequency of population-specific neutral variants. The introduction of patients’ and control genotypes with associated phenotypes into a nationwide database currently being created will simplify the interpretation of variants, which remains the main challenge at present. In general, NGS-based analyses result in an increased number of incidental findings or variants of unknown significance. The patient must be informed about this possibility before the testing and must have the opt in / opt out possibility clearly formulated in the informed consent. Consensus on what incidental information should be disclosed has yet to be reached. Currently, there is general agreement on reporting mutations in known high-penetrant genes in patients with a typical personal and family cancer history [38]. However, there is no agreement on pathogenic mutations in genes with lower penetrance or on mutations related to autosomal-recessive syndromes. These questions are currently being tackled in cooperating centers on a rather individual basis, depending on the formulation of the informed consents obtained, and on the clinical experience of the indicating geneticists [49].

In conclusion, CZECANCA allows comprehensive testing for a majority of frequent hereditary cancer syndromes while mitigating potential difficulties of incidental findings in non-cancer genes as seen in exome or genome sequencing. The reliability of the procedure enables an unbiased identification of variants present in patients, which together with a correct interpretation of variants is key for the effective management of hereditary cancer patients and their relatives.

## Supporting information

S1 Table List of 219 CZECANCA targeted genes with basic characteristics of their protein products. The primary gene target for the probe coverage was represented by coding sequences (cds) representing all exons (in case of known cancer susceptibility genes) or all coding exons (in other genes), including 10 bases from adjacent intronic regions. The promoter regions of the *BRCA1* and *BRCA2* genes were included into the primary target. Because of the strict design conditions, some clinically important regions were left untargeted (highlighted) for technical reasons such as repeats and homologous regions. (The characteristics of protein products were obtained from string.embl.de and/or genecards.org).
(XLSX)

S2 Table Regions of interest with low coverage ≤20X. The average coverage is the mean from 10 randomly selected samples.
(XLSX)

S3 Table Comparison of identified variants in the targeted exonic regions and 12 bp from adjacent introns with GATK quality >100 in three intra-run replicates of sample #2268. The DNA sample pooled for the enrichment in amounts corresponding to 33 ng (e.g. 1/30; considered as 100%), 75% and 50% of this amount, respectively. (Cov = coverage; Q = quality; discordant variants are highlighted).
(XLSX)

S4 Table Comparison of identified variants in the targeted exonic regions and 12 bp from adjacent introns with GATK quality >100 in two independent run replicates of sample #3647. All values of coverages (Cov) of sample #3647 in run 14 were corrected by a factor of 1.3880 to normalize coverages between samples for presentation in Fig 4B. (Q = quality; discordant variants are highlighted).
(XLSX)

S5 Table Comparison of identified variants in the targeted exonic regions and 12 bp from adjacent introns with GATK quality >100 in sample #3582 analyzed independently in four participating laboratories(Lab). All values of coverages (Cov) in Lab2, Lab3, and Lab4 were corrected to the coverage of Lab1 by a factor shown in line 336 to normalize coverages between samples for Fig 4C. (discordant variants are highlighted).
(XLSX)

S6 Table List of variants used for the validation of SNVs detection. (XLSX)

S7 Table List of CNVs used for the validation of a large genomic rearrangements analysis. (XLSX)

S8 Table CNV scores (from CNVkit software) of bins in *BRCA1*, *PALB2*, *CHEK2*, and *TP53*. The numbers of samples with previously characterized CNVs are highlighted in red. The table show raw values obtained from CNVkit as well as median-normalized values. The normalized values >0.5 (highlighted in green) were indicative for the presence of a duplication, while values <-0.6 (highlighted in yellow) were indicative for a deletion. Data from this table were used for creation of Fig 5.
(XLSX)

S9 Table Variants identified in five Coriell Institute reference samples sequenced using CZECANCA pipeline and their comparison with VCF files obtained from GIAB website. The considered targeted region encompasses 628,069 bases of CZECANCA target region. False negative variants are highlighted.
(XLSX)

S10 Table Variant consensus analysis report from EMQN (NGS pilot 2016) for CZENCANCA sequencing of a reference sample. (XLSX)

S11 Table Results of CNV analysis performed in two validation sets consisting of four runs from Laboratory 1 (116 samples prepared using the ultrasound DNA fragmentation on Covaris) and four runs from Laboratory 3 (125 other samples prepared using the enzymatic DNA cleavage by Fragmentase). To estimate number of false positive (FP) and false negative (FN) samples, data for CNV analysis of Coriell Institute reference samples (Coriell; 10 samples analyzed in Laboratory 1 and prepared using the ultrasound DNA fragmentation on Covaris) were added. The values in cells represent differences of CNV scores for a given cell (i.e. sample in the coordinate) from the median value of signals from particular sample group (i.e. Coriell—columns Q-Z, Laboratory 1—columns AB-EM, Laboratory 3—columns EO-JI) in a given CNVkit_bin_set_coordinate (column A). Values in cells showing individual analyzed samples from particular sample group exceeding the given CNVkit threshold value for deletion (<-0,6) and duplication (>0,45) are highlighted as red and green cells, respectively. The columns C-O provide several aggregated metrics, that include number of individual samples in which deletion (columns G-I), duplication (J-L), or deletion+duplication (M-O) was found in a given coordinate in particular sample group. Columns C-E enable identification of non-informative bin sets with suspected false positive (FP) signals (indicated by the value = 1) that include regions on X chromosome called in male samples as deletions (highlighted in blue in column B), regions with insufficient coverage or containing pseudogenes (highlighted in orange and yellow, respectively; in column B), or bin sets containing the improbable number of deletions+duplications exceeding the 4% of analyzed samples in a particular sample group.
(XLSX)

S1 Fig Run of consecutive bin set coordinates with values indicating a deletion (< -0.6; red) or a duplication (> 0.45; green) increases the probability of a real rearrangement. The *BRCA1* and *BRIP1* deletions were confirmed by MLPA analyses, which are currently no available for confirmation of secondary findings in *MSR1* or *ZNF350*. (The graphs expressed normalized CNVkit values shown in S11 Table).
(TIF)

S2 Fig CNV analysis of genes *BRCC3*, *FANCB*, *GPC3*, and *UBE2A* localized on X chromosome enabled to demonstrate differences in normalized CNVkit values in samples carrying a real ‘deletion’ in samples prepared by ultrasound DNA fragmentation or enzymatic DNA lysis. The XX and X indicates areas of samples obtained from female and male probands, respectively. (The graphs expressed normalized CNVkit values shown in S11 Table). Upper panel shows normalized CNVkit values in 116 samples analyzed in four runs in laboratory 1. Lower panel shows normalized CNVkit values in 125 other samples analyzed in four runs in laboratory 3.
(TIF)
