# Supplementary figures and images for: Validation of CZECANCA (CZEch CAncer paNel for Clinical Application) for targeted NGS-based analysis of hereditary cancer syndromes
Source: PLoS One. 2018 Apr 12;13(4):e0195761. doi: 10.1371/journal.pone.0195761 (PMC5896995; doi:10.1371/journal.pone.0195761)

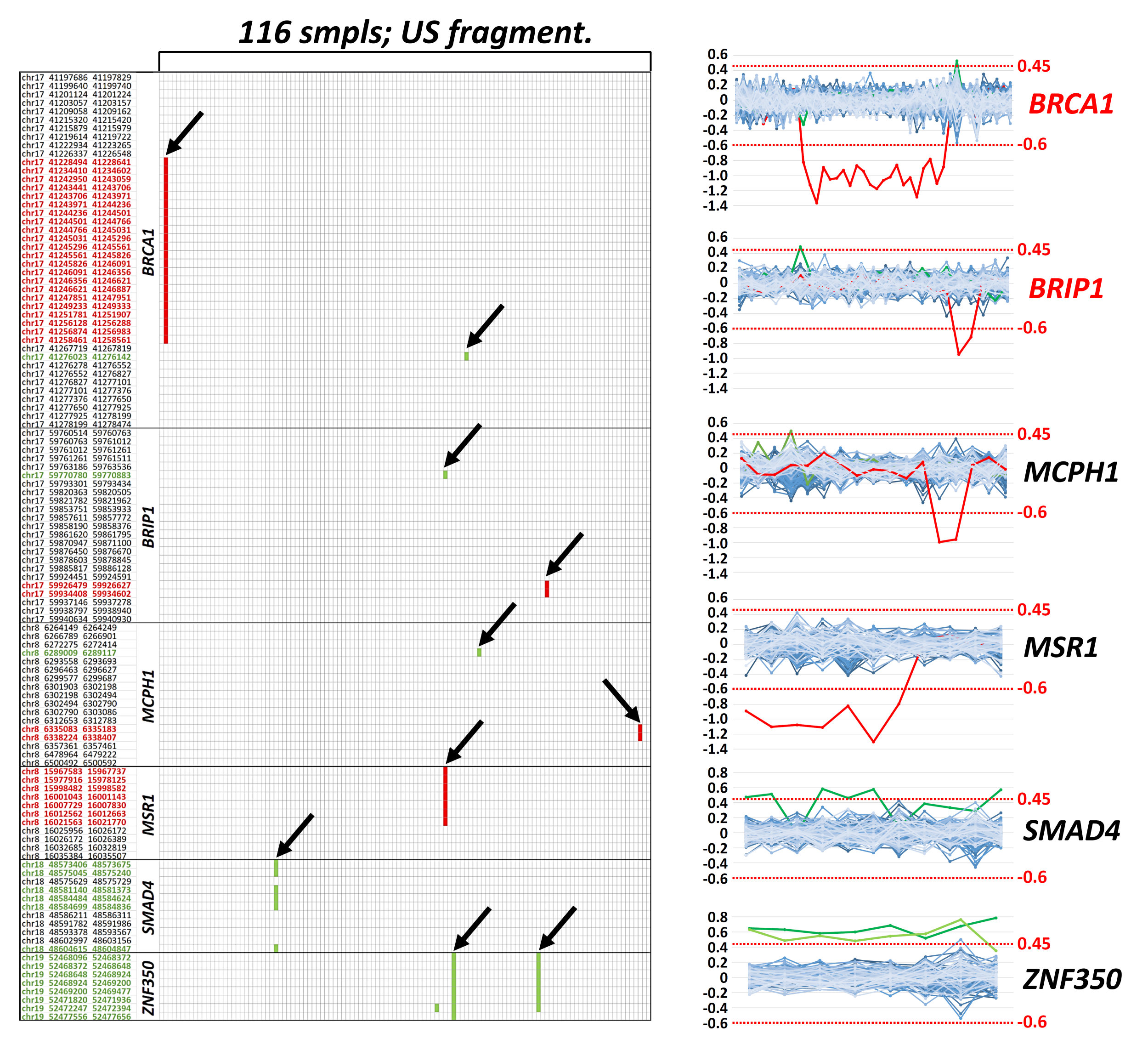

Supplement: S1 Fig — The BRCA1 and BRIP1 deletions were confirmed by MLPA analyses, which are currently no available for confirmation of secondary findings in MSR1 or ZNF350. (The graphs expressed normalized CNVkit values shown in S11 Table). (TIF) [file pone.0195761.s012.tif]

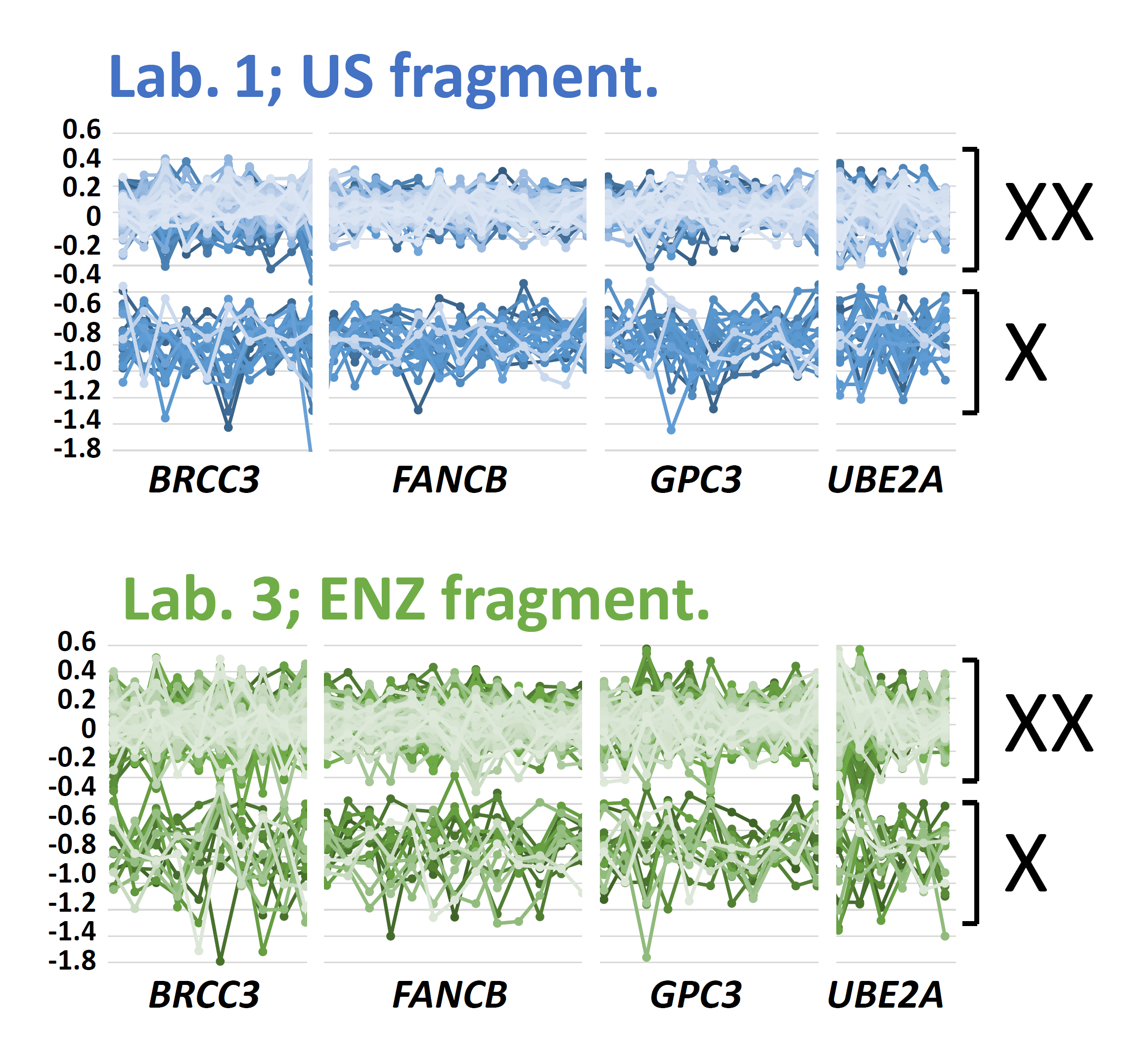

Supplement: S2 Fig — The XX and X indicates areas of samples obtained from female and male probands, respectively. (The graphs expressed normalized CNVkit values shown in S11 Table). Upper panel shows normalized CNVkit values in 116 samples analyzed in four runs in laboratory 1. Lower panel shows normalized CNVkit values in 125 other samples analyzed in four runs in laboratory 3. (TIF) [file pone.0195761.s013.tif]
